# Validity of a point-of-care nerve conduction device for polyneuropathy identification in older adults with diabetes: Results from the Canadian Study of Longevity in Type 1 Diabetes

**DOI:** 10.1371/journal.pone.0196647

**Published:** 2018-04-30

**Authors:** Daniel Scarr, Leif E. Lovblom, Nancy Cardinez, Andrej Orszag, Mohammed A. Farooqi, Genevieve Boulet, Alanna Weisman, Julie A. Lovshin, Mylan Ngo, Narinder Paul, Hillary A. Keenan, Michael H. Brent, David Z. Cherney, Vera Bril, Bruce A. Perkins

**Affiliations:** 1 Lunenfeld-Tanenbaum Research Institute, Mount Sinai Hospital, Toronto, Ontario, Canada; 2 Division of Endocrinology and Metabolism, Department of Medicine, University of Toronto, Toronto, Ontario, Canada; 3 Division of Nephrology, Department of Medicine, University of Toronto, Toronto, Ontario, Canada; 4 The Ellen and Martin Prosserman Centre for Neuromuscular Diseases, Krembil Neuroscience Centre, Division of Neurology, Department of Medicine, University Health Network, University of Toronto, Toronto, Ontario, Canada; 5 Joint Department of Medical Imaging, Division of Cardiothoracic Radiology, University Health Network, Toronto, Ontario, Canada; 6 Research Division, Joslin Diabetes Center, Boston, Massachusetts, United States of America; 7 Department of Ophthalmology and Vision Sciences, Department of Medicine, University of Toronto, Toronto, Ontario, Canada; Ipswich Hospital NHS Trust, UNITED KINGDOM

## Abstract

**Objective:**

Point-of-care nerve conduction devices (POCD) have been studied in younger patients and may facilitate screening for polyneuropathy in non-specialized clinical settings. However, performance may be impaired with advanced age owing to age-related changes in nerve conduction. We aimed to evaluate the validity of a POCD as a proxy for standard nerve conduction studies (NCS) in older adults with type 1 diabetes (T1D).

**Methods:**

Sural nerve amplitude potential (AMP) and sural nerve conduction velocity (CV) was measured in 68 participants with ≥ 50 years T1D duration and 71 controls (from age/sex-matched subgroups) using POCD and NCS protocols. Agreement was determined by the Bland-Altman method, and validity was determined by receiver operating characteristic curves.

**Results:**

T1D were 53% female, aged 66±8yr and had diabetes duration 54yr[52,58]. Controls were 56%(p = 0.69) female and aged 65±8yr(p = 0.36). Mean AMP_POCD_ and CV_POCD_ for the 139 participants was 7.4±5.8μV and 45.7±11.2m/s and mean AMP_NCS_ and CV_NCS_ was 7.2±6.1μV and 43.3±8.3m/s. Mean difference of AMP_POCD_−AMP_NCS_ was 0.3±3.8μV and was 2.3±8.5m/s for CV_POCD_−CV_NCS_. A AMP_POCD_ of ≤6μV had 80% sensitivity and 80% specificity for identifying abnormal AMP_NCS_, while a CV_POCD_ of ≤44m/s had 81% sensitivity and 82% specificity to identify abnormal CV_NCS_. Abnormality in AMP_POCD_ or CV_POCD_ was associated with 87% sensitivity, while abnormality in both measures was associated with 97% specificity for polyneuropathy identification.

**Conclusions:**

The POCD has strong agreement and diagnostic accuracy for identification of polyneuropathy in a high-risk subgroup and thus may represent a sufficiently accurate and rapid test for routinely detecting those with electrophysiological dysfunction.

## Introduction

Polyneuropathy affects up to 50% of people with diabetes and is often underdiagnosed, in part due to a long asymptomatic stage during which identification and management is challenging [1, 2]. It may result in costly clinical sequelae such as pain, loss of balance, ulceration, and amputations, and has been associated with a reduced quality of life [3, 4]. The prevailing concept of the natural history of polyneuropathy involves early, symmetrical, and length dependent injury first to the small nerve fibers of the peripheral nervous system leading to dysfunction of large sensory nerve fibers followed by motor nerve fibers [5–8]. Confirmation of polyneuropathy requires measurement of large fiber dysfunction in two anatomical nerve distributions (frequently the sural and peroneal nerves) demonstrated by reference standard nerve conduction studies (NCS) [9, 10]. This technique involves intensive study in specialized neurology clinics and therefore is generally reserved for symptomatic individuals with later-stage disease in which the etiology is questioned as use of NCS for asymptomatic screening would be limited by cost and the availability of specialized clinics, personnel, and equipment [11, 12]. Simple screening tests that can reasonably rule in or out polyneuropathy for a subset of individuals would fill a major clinical gap by stratifying those that require further diagnostic work-up by an electrophysiological specialist, and enabling early intervention [13, 14].

A novel point-of-care nerve conduction device (POCD) has been developed [15] that has the potential to provide rapid quantification of sensory nerve fiber function and serve as an acceptable proxy to standard NCS. Measures of sural nerve function represent useful indices of polyneuropathy because the etiology of this complication is characterized by a length dependent and initially axonal injury to sensory nerves, with subsequent injury to motor nerves (1, 2). Sural nerve amplitude potential (AMP) and sural nerve conduction velocity (CV) are quantitative measures that reflect the number of axons able to conduct impulses and the relative degree of myelination in the axons, respectively (7). Consequently, these measures of sural nerve function have the greatest face validity as a single parameter for polyneuropathy identification. (1, 7, 11). The novel POCD measures sural nerve amplitude potential (AMP_POCD_) and sural nerve conduction velocity (CV_POCD_) using the same test principles as standard NCS (AMP_NCS_, CV_NCS_), and importantly, it is a rapid test that is easy to administer. It overcomes the need for a specialized technician because the sensor pad surveys a broad area of the lower limb to detect signals from the sural nerve. An earlier version of the device was shown to have strong agreement with NCS measures and it accurately identified polyneuropathy, however its use for clinical practice was limited by device complexity [16, 17]. This device has been evaluated in a cohort of adults with type 1 diabetes (T1D) and type 2 diabetes where it was shown to be a reliable and valid screening instrument to identify polyneuropathy according to standard electrodiagnostic criteria [18]. Although it is widely accepted that normative nerve conduction parameters change with age [19, 20], the diagnostic performance of this device for polyneuropathy identification has not been evaluated in older adults with diabetes.

The Canadian Study of Longevity in Type 1 Diabetes aimed to phenotype neuropathy, nephropathy, and retinopathy in older adults with ≥ 50 years of T1D [21–23] and we had a unique opportunity to evaluate the POCD in this cohort. Considering the exaggerated risk for the development of polyneuropathy in patients with a longer duration of T1D [24], it is important that clinicians are able to objectively identify electrophysiological injury in this high-risk subgroup. Specifically, we aimed to evaluate the novel point-of-care nerve conduction device’s quantitative agreement with reference standard nerve conduction studies, as well as its diagnostic validity for polyneuropathy identification in older adults with longstanding T1D.

## Materials and methods

### Study design

This was a cross-sectional secondary analysis involving participants in the second phase of the Canadian Study of Longevity in Type 1 Diabetes (funded by JDRF, operating Grant No. 17-2013-312). The participants were studied over the course of 2 clinical visits set 2–4 weeks apart, conducted between February 2015 and September 2016. Neurological measurements using the POCD (index test) and standard NCS (reference standard) were performed on the same visit and independent technicians were masked to the results of the other. This manuscript was written in accordance with the Guidelines for Reporting Reliability and Agreement Studies [25] and the Standards for Reporting Diagnostic Accuracy Studies [26].

### Study population

The study population of the second phase of the Canadian Study of Longevity in Type 1 Diabetes consisted of 75 older adults with ≥ 50 years of T1D, and 75 age- and sex-matched controls. Participants with T1D were recruited consecutively from those who took part in the mail-based survey of the first study phase [21–23] and controls were spouses, friends or other family members of T1D participants, or were recruited through community advertisement. Inclusion criteria for T1D participants was ≥ 50 years of diabetes duration and inclusion criteria for controls was sex-matched 1:1 and within 5 years of age of a T1D participant. Inclusion criteria common to both controls and T1D participants was the ability to understand and cooperate with study procedures. Exclusion criteria for controls included: 1) presence of diabetes mellitus, or fasting plasma glucose exceeding 7.0mmol/L; 2) pre-existing kidney disease. Exclusion criteria common to both controls and T1D participants were any current eye infection, corneal damage, severe movement disorder, or proparacaine allergy that could have precluded safe *in vivo* corneal confocal microscopy examination. All participants provided written informed consent, and the study and its procedures were approved by the institutional ethics board at the University Health Network and Mount Sinai Hospital in Toronto, ON, Canada.

### Clinical evaluation and nerve conduction studies (reference standards)

All included participants underwent physical examination, clinical evaluation for medical history, review of medication usage, and neurological evaluation of neuropathic signs and symptoms. The Toronto Clinical Neuropathy Score (TCNS) exam score and Michigan Neuropathy Screening Instrument (MNSI) questionnaire and exam scores were calculated based on these evaluations [27–29]. Standard NCS examinations were carried out using the Counterpoint device (Alpine Biomed Corporation, Fountain Valley, California) according to the standards of the American Association for Neuromuscular and Electrodiagnostic Medicine [30]. A total of 10 sensory and motor parameters from the dominant limb peroneal, tibial, and sural nerves were obtained. Peak-to-peak AMP_NCS_ and CV_NCS_ were measured at a fixed distance of 140 mm. Peroneal nerve amplitude potential, conduction velocity, and F wave latency were also measured. All sensory nerve conduction results were acquired following antidromic stimulation of the nerve. Stimulating probes were placed according to the discretion of the trained technician and limbs were maintained above 32°C. Standard NCS measures were obtained over a 2-minute period for each patient. We note that NCS measures were obtained while T1D participants were undergoing euglycemic clamp procedures, where, for purposes of renal hemodynamic function testing, blood glucose was measured every 15 minutes and insulin infusion was adjusted to maintain glucose between 4 and 6 mmol/L for 4h.

We used the following four reference standard definitions: 1) Abnormal sural nerve amplitude, defined by AMP_NCS_ value ≤7.2μV for patients less than or equal to 65 years old and ≤5.5μV for patients older than 65 years old;[31] 2) Abnormal sural nerve conduction velocity, defined by CV_NCS_ value ≤40m/s;[31] 3) the Toronto consensus criteria of the American Academy of Neurology, the American Academy of Electrodiagnostic Medicine, and the American Academy of Physical Medicine and Rehabilitation, defined by the presence of one or more abnormal reference standard NCS parameters in both the sural nerve and peroneal nerve of the dominant lower limb, corroborated by the presence of a clinical sign and/or symptom of neuropathy;[9, 10] and 4) a modified electrophysiology-based Toronto consensus, defined by the presence of one or more abnormal reference standard NCS parameters in both the sural nerve and peroneal nerve of the dominant lower limb.

### Point-of-care nerve conduction device (index test)

Participants were examined bilaterally on the lower limbs using the POCD (DPN-Check, Neurometrix Inc., Waltham, MA) [15]. Examinations were completed by a technician without prior standard NCS training. The device consisted of a single handheld unit that allowed for placement of a disposable biosensor at a distance of 92.2 mm from the stimulation probes located at the opposite end of the device. The biosensor covered a wide area of the limb, such that nerve conduction measures were captured without the need for specialized personnel. The device contained an infrared thermometer located below the stimulating probes to detect limb temperature. The device adjusted for skin temperatures between 23°C and 30°C, and prevented tests from beginning when ankle temperatures were below 23°C.

The lower limbs were prepared using a sterile pad which also buffered the testing area. The stimulating probes were coated in a gel to promote conduction of the impulse generated by the probes. The largest probe was placed on the lateral side of the ankle over the anatomical position of the sural nerve, anterior to the achilles tendon, and posterior to the lateral malleolus. The medial edge of the biosensor was placed on the lower calf in line with a proximal trajectory of the achilles tendon. Once the device was in place, the test was initiated once the start button on the device was activated by the technician. The sural nerve was stimulated between 4–16 times within 10 seconds and the number of stimulation signals was dependent on the strength of the sural nerve signal as detected by the biosensor. If a device error was observed on the display screen, the testing protocol was repeated. The procedure took approximately 2 minutes for each participant. To evaluate the agreement and diagnostic validity of the POCD in comparison to standard NCS, dominant limb AMP_POCD_ and CV_POCD_ measures were used as the index tests.

The prevailing concept of diabetic polyneuropathy indicates that nerve injury occurs symmetrically [5, 6], and therefore we quantified the reliability of the POCD to measure sural nerve function in both the dominant and non-dominant lower limbs using bilateral AMP_POCD_ and CV_POCD_ measures: the POCD demonstrated acceptable reliability for measurement in the left and right lower limbs, with intraclass correlation coefficient (ICC) class (2,1) values [32] of 0.77 for AMP_POCD_ and 0.70 for CV_POCD_.

### Statistical analysis

Analyses were performed using SAS version 9.2 for Windows (SAS Institute, Cary, NC, USA). Clinical characteristics of the T1D and control groups were compared using Student’s t-test for normally distributed variables, the Wilcoxon rank-sum test for non-normally distributed variables, or the χ^2^-test for frequencies. Agreement was calculated as the arithmetic mean of the difference between AMP_POCD_ and AMP_NCS_ and between CV_POCD_ and CV_NCS_ (expressed as POCD values minus NCS values) using the method of Bland and Altman and 95% limits of agreement [33]. Diagnostic validity of the POCD was determined in the T1D participants and was analyzed using two approaches: 1) receiver operating characteristic (ROC) curves, and 2) an algorithm-based protocol. First, ROC curves were generated to determine the area under the curve (AUC) and the optimal thresholds for AMP_POCD_ and CV_POCD_ to identify normal and abnormal AMP_NCS_ and CV_NCS_, respectively (reference standard definitions 1 and 2). Using the optimal thresholds for AMP_POCD_ and CV_POCD_ determined above, we identified participants as having 0, 1, or 2 abnormal POCD results, and then generated ROC curves for this new variable to identify neuropathy based on the modified Toronto consensus (reference standard definition 4). Optimal thresholds were determined by finding the point on the ROC curve closest to the point of perfect discrimination using the formula $\sqrt{{\left(0-x\right)}^{2}+{\left(1-y\right)}^{2}}$. The second approach’s protocol was developed according to the following algorithm: two threshold values were sought for each of AMP_POCD_ and CV_POCD_, one to maximize sensitivity and the other to maximize specificity, such that the negative likelihood ratio would approach 0.1, while the positive likelihood ratio would approach 10; this model was used to test the performance of the POCD in a clinical screening setting[34], and it used the Toronto consensus as the outcome (reference standard definition 3). An α-level of 0.05 was used for tests of statistical significance.

Undetectable CV_NCS_ was assigned a value of 28.9m/s and undetectable CV_POCD_ was assigned a value of 22m/s, the lowest values in the data set, respectively. The planned sample size of 75 T1D participants was based on the renal hemodynamic primary outcomes of the second phase of the study; for the diagnostic validity aspect of this analysis, our sample size of 139 had >99% power to discriminate a conservatively-modeled AUC of 0.75 from the null hypothesis in which the diagnostic accuracy is no different than chance alone (AUC = 0.5) [35].

## Results

**Fig 1:**
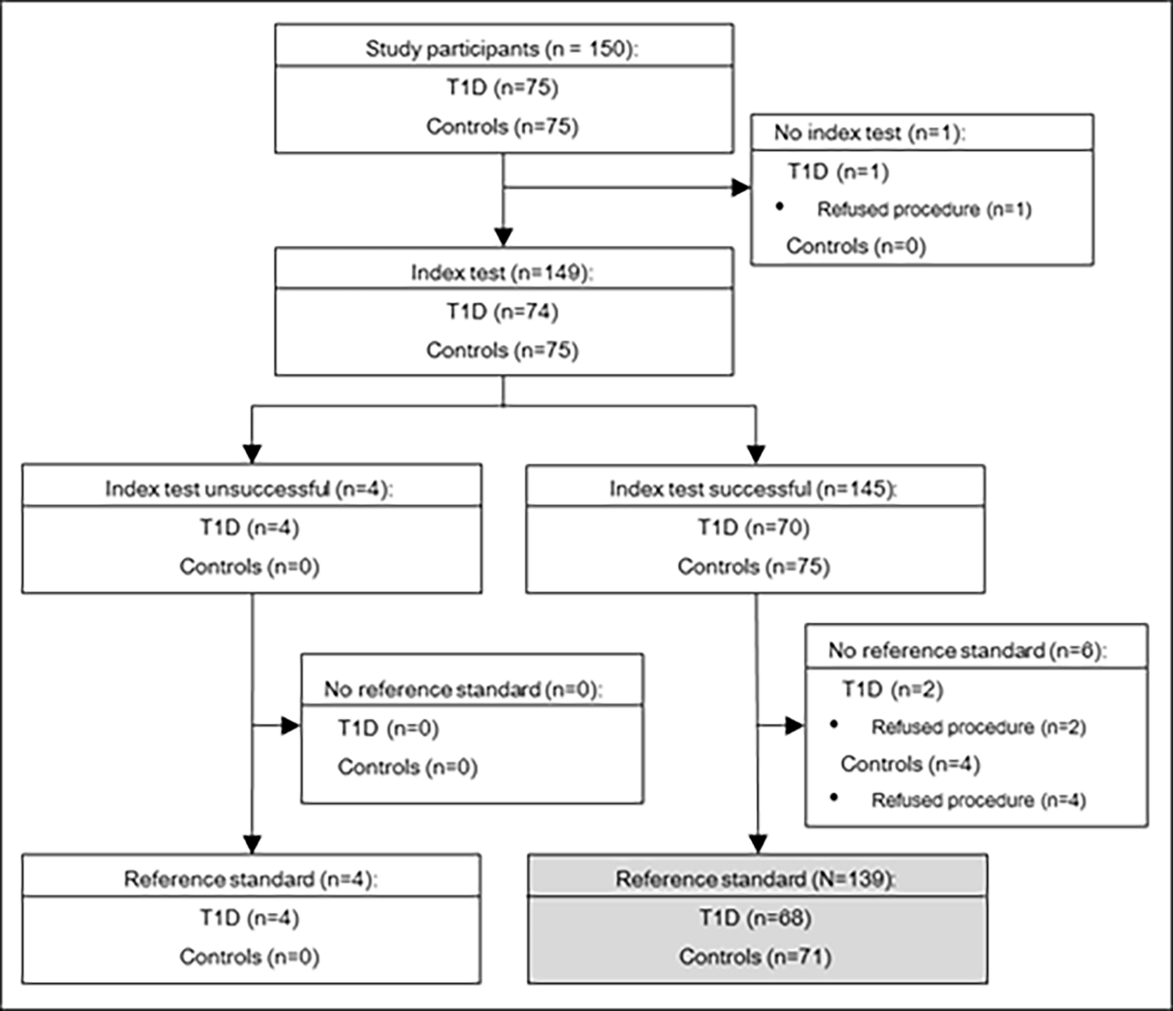
Flow of study participants. Of the 150 study participants in the Canadian Study of Longevity in Type 1 Diabetes, 1 refused the POCD procedure (index test) and 6 refused the NCS procedure (reference standard). Four study participants were excluded due to device errors using the POCD, resulting in 139 participants for analysis.

Among eligible participants enrolled in the study, 68/75 (91%) T1D participants and 71/75 (95%) controls underwent both the index test and reference standard (N = 139, **Fig 1**). Controls and T1D participants included in this study had similar age (65±8 v. 66±8 years, p = 0.36) and the proportion of participants who were of female sex was similar (56 v. 53%, p = 0.69). T1D participants had median diabetes duration of 54[52,58] years. Average HbA1c was 5.6±0.4% (38±4.4 mmol/mol) for controls and 7.3±0.8% (56±8.7 mmol/mol) for T1D participants (p<0.001). Controls had lower median TCNS total (2[0,4] v. 6[4,9], p<0.001), MNSI exam score (2[0.5,3] v. 3[1,4.5], p<0.001), and MNSI questionnaire score (1[0,2] v. 2[0,3], p = 0.032). According to TCNS, 35(52%) participants with T1D had neuropathy. According to the MNSI exam, 39/68 (57%) participants with T1D had neuropathy (score ≥ 2.5). Based on standard electrophysiological testing, 60 (88%) participants with T1D had abnormal AMP_NCS_ and 47 (69%) had abnormal CV_NCS_. Other key clinical and biochemical characteristics are presented in **Table 1**.

**Table 1:** Baseline characteristics for the 139 participants.

Characteristic	Controls (n = 71)	T1D (n = 68)	p-value
*Clinical Characteristics*			
Female sex, n (%)	40 (56)	36 (53)	0.69
Age (years)	65±8	66±8	0.36
Diabetes duration (years)	-	54 [52,58]	-
Age at onset (years)	-	10 [5,17]	-
Daily insulin dose (units/kg)	-	0.48±0.1	-
Height (m)	1.7±0.1	1.6±0.1	0.22
Weight (kg)	76.2±16.2	72.4±12.0	0.11
Body mass index (kg/m^2^)	27.4±5.5	26.7±3.8	0.35
Systolic blood pressure (mmHg)	129±18	133±16	0.11
Diastolic blood pressure (mmHg)	79±9	71±9	<0.001
Resting heart rate (bpm)	67±9	70±11	0.088
*Biochemical Characteristics*			
HbA_1c_ (%)	5.6±0.4	7.3±0.8	<0.001
HbA_1c_ (mmol/mol)	38±4.4	56±8.7	<0.001
Total cholesterol (mmol/L)	4.8±1.0	3.9±0.8	<0.001
LDL cholesterol (mmol/L)	2.8±0.8	1.9±0.5	<0.001
HDL cholesterol (mmol/L)	1.4±0.4	1.7±0.5	<0.001
Triglycerides (mmol/L)	1.5±1.0	0.8±0.4	<0.001
Creatinine (μmol/L)	71.9±12.7	84.4±23.0	<0.001
Urine ACR (mg/mmol)	1.0 [0.6,2.2]	1.5 [0.9,6.0]	0.053
Albumin excretion > 30 mg/day, n (%)	3 (4)	15 (22)	0.002
GFR_INULIN_ (mL/min/1.73m^2^)	102±23	99±25	0.47
*Clinical Neuropathy Scales*			
TCNS total (out of 19)	2 [0,4]	6 [4,9]	<0.001
MNSI exam (out of 10)	2 [0.5,3]	3 [1,4.5]	<0.001
MNSI questionnaire (out of 15)	1 [0,2]	2 [0,3]	0.032
*Neuropathy Outcomes*			
TCNS>5	11 (15%)	35 (52%)	<0.001
MNSI exam score≥2.5	28 (39%)	39 (57%)	0.035
Abnormal AMP_NCS_	15 (21%)	60 (88%)	<0.001
Abnormal CV_NCS_	1 (1%)	47 (69%)	<0.001
Neuropathy by modified Toronto consensus	11 (15%)	62 (91%)	<0.001
Neuropathy by Toronto consensus	8 (11%)	60 (88%)	<0.001

T1D, type 1 diabetes; ACR, albumin to creatinine ratio; GFR_INULIN_, measured glomerular filtration rate; TCNS, Toronto Clinical Neuropathy Score; MNSI, Michigan Neuropathy Screening Instrument; AMP, sural nerve amplitude potential; CV, sural nerve conduction velocity. Data are means ± SD, median [IQR], or n(%).

**Fig 2:**
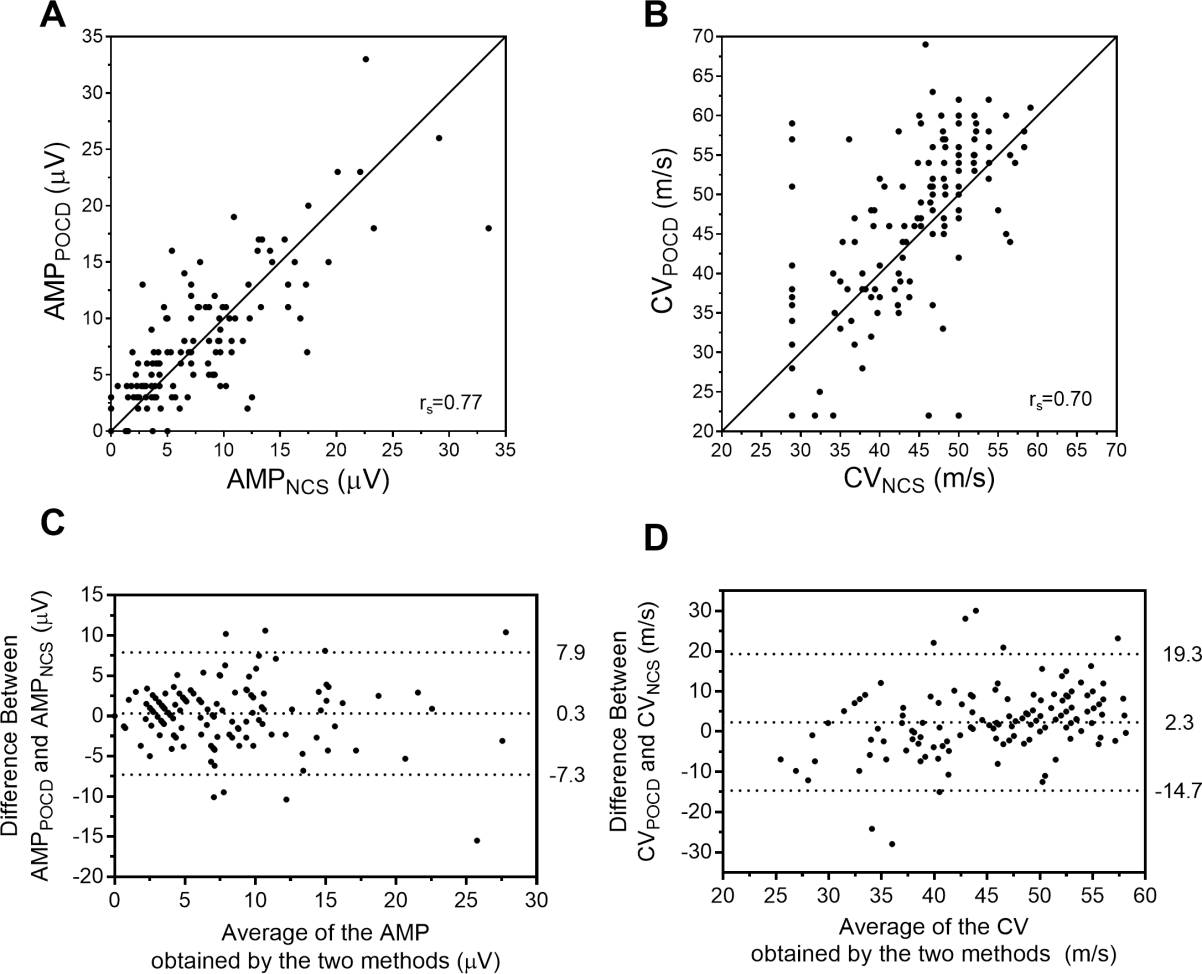
Scatterplots (A, B) and Bland-Altman plots (C, D) for comparison of AMP and CV as determined by POCD and standard NCS. Panels A and B display the scatterplot of AMP (A) and CV (B) obtained by the two methods, r_s_ refers to Spearman’s rank correlation coefficient, and the solid diagonal line represents the line of unity (x = y). Panels C and D display the Bland-Altman plots demonstrating the difference between AMP_POCD_−AMP_NCS_ (C) and CV_POCD_−CV_NCS_ (D); points above or below zero on the y-axis represent overestimation and underestimation by the POCD, respectively. The dotted lines (C, D) correspond to, from top to bottom, the 97.5^th^ percentile of differences, the mean difference, and the 2.5^th^ percentile of differences.

The quantitative measures of AMP_POCD_ and CV_POCD_ are reported in **Table 2** and are also depicted graphically using scatterplots and Bland-Altman plots in **Fig 2**. Compared to controls, T1D had lower AMP_NCS_ (11.0±5.9 v. 3.1±2.9μV, p<0.001), CV_NCS_ (49.7±4.1 v. 36.9±6.2m/s, p<0.001), AMP_POCD_ (10.4±6.2 v. 4.4±3.2μV, p<0.001) and CV_POCD_ (50.9±8.0 v. 40.3±11.5m/s, p<0.001). Mean difference [95% limits of agreement] for the 139 study participants was 0.3±3.8μV [−7.3,7.9μV] between AMP_POCD_−AMP_NCS_ and was 2.3±8.5m/s [−14.7,19.3m/s] between CV_POCD_−CV_NCS._ Mean difference between AMP_POCD_−AMP_NCS_ was −0.6±4.8μV for controls and was 1.2±2.1μV in the T1D subgroup (p = 0.003). Mean difference between CV_POCD_−CV_NCS_ was 1.2±7.2m/s for controls and was 3.5±9.5m/s in the T1D subgroup (p = 0.11).

**Table 2:** Quantification of AMP and CV using standard NCS and the POCD.

Characteristic	Study Population (N = 139)	Controls (n = 71)	T1D (n = 68)	p-value
*Sural nerve amplitude potential*				
AMP_POCD_ (μV)	7.4±5.8	10.4±6.2	4.4±3.2	<0.001
AMP_NCS_ (μV)	7.2±6.1	11.0±5.9	3.1±2.9	<0.001
Correlation*	0.77	0.60	0.73	-
Difference (μV)^†^	0.3±3.8	−0.6±4.8	1.2±2.1	0.003
95% limits of agreement	[−7.3,7.9]	[−10.2,9]	[−3,5.4]	-
*Sural nerve conduction velocity*				
CV_POCD_ (m/s)	45.7±11.2	50.9±8.0	40.3±11.5	<0.001
CV_NCS_ (m/s)	43.4±8.3	49.7±4.1	36.9±6.2	<0.001
Correlation*	0.70	0.60	0.67	-
Difference (m/s)^‡^	2.3±8.5	1.2±7.2	3.5±9.5	0.11
95% limits of agreement	[−14.7,19.3]	[−13.2,15.6]	[−15.5,22.5]	-

AMP, sural nerve amplitude potential; CV, sural nerve conduction velocity; NCS, nerve conduction studies; POCD, point-of-care nerve conduction device; T1D, type 1 diabetes. Data are means±SD, unless otherwise indicated. P-value for comparison of the control and T1D subgroups.

*Spearman correlation coefficients between POCD and NCS measures.

†Expressed as AMP_POCD_−AMP_NCS_.

‡Expressed as CV_POCD_−CV_NCS_.

**Fig 3:**
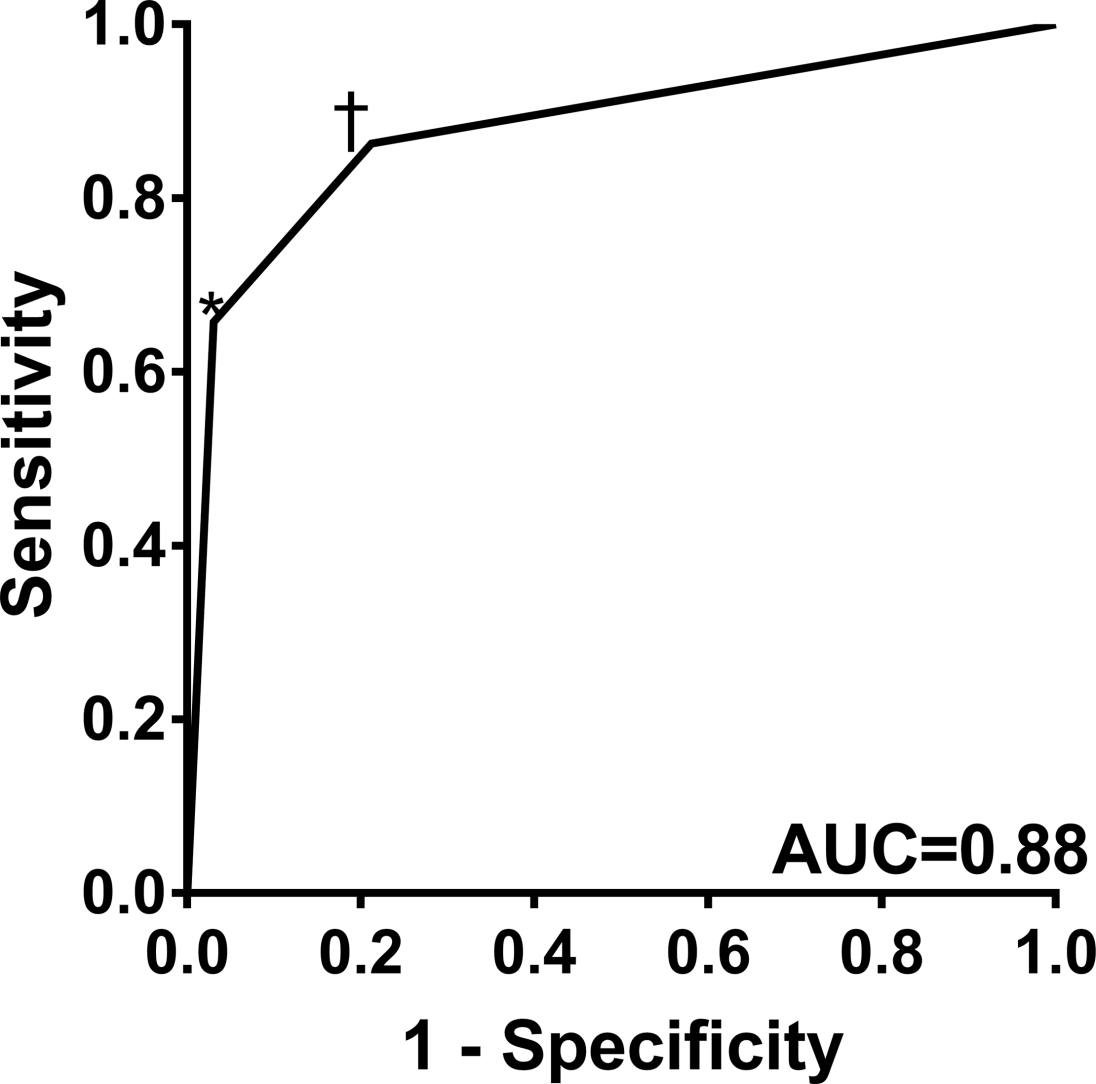
ROC curve displaying the diagnostic validity of the POCD for polyneuropathy identification as defined by electrodiagnostic standard NCS. An optimal threshold of one abnormality in AMP_POCD_ or CV_POCD_ (†) had a sensitivity and specificity of 86% and 79%, respectively. An optimal threshold of abnormality in AMP_POCD_ and CV_POCD_ (*) had a sensitivity and specificity of 66% and 97%, respectively.

Due to the presence of measurement differences, ROC curves were generated to obtain threshold values using the POCD to identify abnormal age-adjusted NCS values. A AMP_POCD_ of ≤6μV had 80% sensitivity and 80% specificity for identifying abnormal AMP_NCS_, while a CV_POCD_ of ≤44m/s had 81% sensitivity and 82% specificity for identifying abnormal CV_NCS_. Using the derived AMP_POCD_ and CV_POCD_ optimal thresholds, we aimed to quantify the diagnostic performance of the POCD, specifically to determine whether abnormalities in AMP_POCD_ and/or CV_POCD_ could identify polyneuropathy based on the modified Toronto consensus. This ROC curve analysis (**Fig 3**) showed that 1) abnormality in either AMP_POCD_ or CV_POCD_ had a sensitivity of 86% and specificity of 79%, and 2) abnormality in both AMP_POCD_ and CV_POCD_ had a sensitivity of 66% and specificity of 97%.

To evaluate the performance of this device in a clinical model, we sought two additional thresholds for each of AMP_POCD_ and CV_POCD_−one that maximizes sensitivity while the other maximizes specificity. A AMP_POCD_ of ≤9μV had 92% sensitivity, 58% specificity, and a negative likelihood ratio of 0.14 to detect abnormal AMP_NCS_, while a AMP_POCD_ of ≤3μV had 48% sensitivity, 92% specificity, and a positive likelihood ratio of 6. A CV_POCD_ of ≤48m/s had 90% sensitivity, 66% specificity, and a negative likelihood ratio of 0.15 to detect abnormal CV_NCS_, while a CV_POCD_ of ≤37m/s had 52% sensitivity, 92% specificity, and a positive likelihood ratio of 6.5. Using the specific and sensitive thresholds, we tested a clinical model where 1) patients with a AMP_POCD_ of ≤3μV or CV_POCD_ of ≤37m/s were classified as having polyneuropathy, 2) those with a AMP_POCD_ of >9μV or CV_POCD_ of >48m/s were classified as not having polyneuropathy, and 3) those with a AMP_POCD_ between 4–9μV or CV_POCD_ between 38-48m/s would not be classified and subsequently referred for NCS follow up to confirm the presence or absence of polyneuropathy. This algorithm had a sensitivity of 85% and specificity of 92% for polyneuropathy identification, and (25) 18% of participants were not classified such that in a clinical model they would be referred for standard NCS.

## Discussion

In this cross-sectional analysis of older adults with ≥ 50 years of T1D, we found that a novel point-of-care nerve conduction device can be used as a rapid electrophysiological proxy for standard nerve conduction studies. There was a very low magnitude of systematic error (or measurement bias) between the POCD and standard NCS–specifically, the device overestimated sural nerve amplitude potential by 1.2μV and overestimated sural nerve conduction velocity by 3.5m/s, each representing less than a five percent mean bias. We were able to accurately determine POCD-specific threshold values that serve to identify abnormal levels of sural nerve amplitude potential and sural nerve conduction velocity as determined by standard NCS. Furthermore, we confirmed that the device was able to accurately identify polyneuropathy, according to the reference standard research definition, using these POCD-specific thresholds. Finally, we demonstrated the proof-of-concept that a clinical algorithm which implemented more sensitive and specific threshold values from the point-of-care device could classify polyneuropathy status for a majority of participants (82%) with strong sensitivity and specificity, leaving an acceptable proportion (18%) that was left unclassified and for who further testing or follow-up could be considered.

The POCD was able to identify abnormality in standard NCS parameters with strong operating characteristics. Using ROC curve analysis, the POCD identified abnormality in standard NCS with optimal thresholds of ≤6μV (80% sensitivity and 80% specificity) for AMP_POCD_ and ≤44m/s (81% sensitivity and 82% specificity) for CV_POCD_. The device identified polyneuropathy with 86% sensitivity and 79% specificity for abnormality in either AMP_POCD_ or CV_POCD_ and 66% sensitivity and 97% specificity for a more specific definition comprising abnormality in both AMP_POCD_ and CV_POCD_. Our findings suggest that optimized thresholds for abnormality in POCD measures can be used to diagnose polyneuropathy.

While there are a limited number of studies which have evaluated this device [16–18, 36, 37], one study [18] showed that this device was a reliable and valid tool for polyneuropathy identification and reported thresholds of ≤6μV for AMP_POCD_ and ≤48m/s for CV_POCD_ in 44 adults with T1D and type 2 diabetes. Comparably, we found that the diagnostic performance and POCD-specific thresholds in older adults with longstanding T1D –despite any age-related changes in nerve conduction–were similar to those reported in younger adults with diabetes, implying that this device can be used as a valid screening test for polyneuropathy across broadly-aged adult populations. Though we report specific threshold values, we acknowledge that these require further confirmation using point-of-care nerve conductions for polyneuropathy identification in future studies implemented in a variety of clinical settings. Furthermore, we found a small but statistically significant difference in agreement in sural amplitude between T1D and control participants, which was associated with controls having significantly higher AMP_NCS_ values. This difference does not affect our interpretation of the diagnostic validity analyses, but users of the PCOD should be aware of a potential overestimation by the POCD when examining patients with inherently lower NCS values.

Given the limitations to the feasibility of the widespread use of standard NCS in routine clinical screening for neuropathy [9], we evaluated the ability of the POCD to fill a care gap by rapidly classifying a majority of patients with polyneuropathy while minimizing the proportion that could not be confidently classified by the algorithm. This was done using a model that considers two separate diagnostic thresholds, one that maximizes sensitivity (and the negative likelihood ratio) and another that maximizes specificity (and the positive likelihood ratio) for AMP_POCD_ and CV_POCD_. The device classified 82% of the participants with a sensitivity of 85% and specificity of 92%, while 18% were left unclassified, implying that such patients in a clinical setting could be referred for further specialist evaluation, could receive further testing after an interval of time when their classification may become more certain, or in research settings could be considered to be at higher risk of subsequent polyneuropathy onset. Using this model approach [34, 38], we propose the existence of an efficient triage protocol that permits simple and rapid neuropathy classification in the vast majority of subjects. Akin to the thresholds determined for each point-of-care device parameter, such an algorithm will require further validation in diverse populations and settings.

A simple generalizable test, such as the POCD, has the potential to fill a gap in clinical care and in clinical research protocols. Peripheral neuropathies have a long and latent sub-clinical phase, where up to 50% of cases have been estimated to be asymptomatic and patients often undergo specialist evaluation using NCS to confirm polyneuropathy upon the presence of clinical neuropathic signs or symptoms [1]. Current neuropathy screening guidelines recommend screening through physical examination maneuvers such as the 10g monofilament test and the 128Hz vibration tuning fork test [1, 39]; however the POCD provides a rapid, reliable, valid, and more objectively quantifiable measure of detecting electrophysiological abnormality as compared to physical examination tests [40]. Routine assessment of electrophysiological abnormality may be useful in quantitatively identifying asymptomatic stages of polyneuropathy. The ideal strategy for intervention would be to identify polyneuropathy early in its course, during an asymptomatic phase, and to apply a successful disease-modifying therapy for prevention of the onset of clinical manifestations [2]. This strategy has been hindered by the lack of an early biomarker. Consequently, adjustments to lifestyle and pain management agents remain as the primary therapeutic options for patients with polyneuropathy [1]. Earlier identification of electrophysiological abnormality using rapid generalizable screening protocols may invoke better recognition for follow up and therapy for those at risk.

Our findings indicate that the POCD has acceptable accuracy, strong diagnostic performance, and could provide a simple and rapid method for nerve conduction measures for use in routine screening of polyneuropathy, such as in a diabetes complications screening clinic. However, we acknowledge the presence of limitations in this study. First, T1D participants were included only if they had ≥ 50 years of diabetes. Such selection may bias results toward weaker diagnostic performance. Second, the current study population had a high prevalence of polyneuropathy (88%), which may not reflect that seen in the general practice reference population. However, we note that common trials and cohort studies suggest that incidence of polyneuropathy in longstanding T1D is sufficiently high to invoke high prevalence in this age group [24]. Third, although the initiating injury to large nerve fibers in polyneuropathy is thought to occur to the sensory nerve fibers diffusely, the POCD device is specific to sural nerve function. Fourth, we used a non-neurologist technician to conduct the testing protocol using the POCD in order to evaluate generalizability to non-specialty clinics, and therefore stronger agreement and validity against NCS may be observed in settings that employ highly specialized personnel to conduct the index test. We acknowledge that our NCS testing protocols were different than general standard procedure in that they were part of a panel of other deep-phenotyping tests performed during euglycemic clamp. We could not determine the influence of euglycemia on the distribution of NCS results, but we see this standardization as a potential strength of this diagnostic accuracy study. Finally, our repeatability analysis was restricted to intra-rater comparisons only (rather than inter-rater). Estimates of inter-rater reliability in younger populations showed ICCs of approximately 0.80.[18]

This analysis of the Canadian Study of Longevity in Type 1 Diabetes cohort aimed to evaluate the diagnostic performance of a POCD as a rapid screening test for polyneuropathy identification in older adults with ≥ 50 years of type 1 diabetes. We confirmed its ability to detect electrophysiological abnormalities as measured using standard NCS. By applying a triage model which used both sensitive and specific thresholds for electrophysiological abnormality we demonstrated the concept that this device can be used as a rapid and applicable screening test for polyneuropathy identification.

## Supporting information

S1 File Supporting data. (XLSX)
